# The Promise and Pitfalls of Using Crowdsourcing in Research Prioritization for Back Pain: Cross-Sectional Surveys

**DOI:** 10.2196/jmir.8821

**Published:** 2017-10-06

**Authors:** Matthew A Bartek, Anjali R Truitt, Sierra Widmer-Rodriguez, Jordan Tuia, Zoya A Bauer, Bryan A Comstock, Todd C Edwards, Sarah O Lawrence, Sarah E Monsell, Donald L Patrick, Jeffrey G Jarvik, Danielle C Lavallee

**Affiliations:** ^1^ Surgical Outcomes Research Center Department of Surgery University of Washington Seattle, WA United States; ^2^ Comparative Effectiveness, Cost and Outcomes Research Center Department of Radiology University of Washington Seattle, WA United States; ^3^ Center for Biomedical Statistics Department of Biostatistics University of Washington Seattle, WA United States; ^4^ Department of Heath Services School of Public Health University of Washington Seattle, WA United States

**Keywords:** research prioritization, crowdsourcing, MTurk, Amazon Mechanical Turk, patient engagement, stakeholder engagement, back pain, comparative effectiveness research, patient participation, low back pain

## Abstract

**Background:**

The involvement of patients in research better aligns evidence generation to the gaps that patients themselves face when making decisions about health care. However, obtaining patients’ perspectives is challenging. Amazon’s Mechanical Turk (MTurk) has gained popularity over the past decade as a crowdsourcing platform to reach large numbers of individuals to perform tasks for a small reward for the respondent, at small cost to the investigator. The appropriateness of such crowdsourcing methods in medical research has yet to be clarified.

**Objective:**

The goals of this study were to (1) understand how those on MTurk who screen positive for back pain prioritize research topics compared with those who screen negative for back pain, and (2) determine the qualitative differences in open-ended comments between groups.

**Methods:**

We conducted cross-sectional surveys on MTurk to assess participants’ back pain and allow them to prioritize research topics. We paid respondents US $0.10 to complete the 24-point Roland Morris Disability Questionnaire (RMDQ) to categorize participants as those “with back pain” and those “without back pain,” then offered both those with (RMDQ score ≥7) and those without back pain (RMDQ <7) an opportunity to rank their top 5 (of 18) research topics for an additional US $0.75. We compared demographic information and research priorities between the 2 groups and performed qualitative analyses on free-text commentary that participants provided.

**Results:**

We conducted 2 screening waves. We first screened 2189 individuals for back pain over 33 days and invited 480 (21.93%) who screened positive to complete the prioritization, of whom 350 (72.9% of eligible) did. We later screened 664 individuals over 7 days and invited 474 (71.4%) without back pain to complete the prioritization, of whom 397 (83.7% of eligible) did. Those with back pain who prioritized were comparable with those without in terms of age, education, marital status, and employment. The group with back pain had a higher proportion of women (234, 67.2% vs 229, 57.8%, *P*=.02). The groups’ rank lists of research priorities were highly correlated: Spearman correlation coefficient was .88 when considering topics ranked in the top 5. The 2 groups agreed on 4 of the top 5 and 9 of the top 10 research priorities.

**Conclusions:**

Crowdsourcing platforms such as MTurk support efforts to efficiently reach large groups of individuals to obtain input on research activities. In the context of back pain, a prevalent and easily understood condition, the rank list of those with back pain was highly correlated with that of those without back pain. However, subtle differences in the content and quality of free-text comments suggest supplemental efforts may be needed to augment the reach of crowdsourcing in obtaining perspectives from patients, especially from specific populations.

## Introduction

Modern health care decision making incorporates expert opinion, practice standards, and the individual preferences and values of patients themselves [1,2]. The patient’s voice is essential to ensuring that treatment plans address what is most important to them. In support of patient-centered care, patient-centered outcomes research equally seeks to engage patients and the public in designing and implementing research studies. Efforts to involve patients in research can take various forms ranging from consultative (eg, researchers can seek patient opinion about the design of a study) to more collaborative approaches (eg, patients can be involved as members of the study team itself). Engagement throughout the research process is an important step in developing evidence that will support patients and providers as they make health care decisions. Identifying and prioritizing research topics—the first phases of patient-centered outcomes research—direct researchers to address the relevant and important problems facing those who may benefit most from study findings; thus, patient involvement is imperative [3].

Patient-centered outcomes research teams have begun to use novel technology-driven engagement strategies—including social media and crowdsourcing platforms—to augment traditional engagement activities. Emerging evidence has suggested that online engagement methods such as crowdsourcing may provide an efficient alternative to in-person meetings [4,5]. Crowdsourcing as a whole is appealing in its ability to rapidly obtain responses from a broad and potentially diverse population. For prevalent conditions, such platforms may provide an efficient and effective method for obtaining input on research activities, including research prioritization.

One example is Amazon Mechanical Turk (MTurk; Amazon.com, Inc), a crowdsourcing platform where users are paid a small fee for performing designated tasks [6]. Originally designed to allow the rapid completion of complex but repetitive work, MTurk has been adopted by behavioral scientists and market researchers to serve as a virtual laboratory to quickly and inexpensively administer thought experiments via online surveys, perform market research for organizations, and give insight into the thought processes underlying decision making [4,7-9]. Furthermore, some have begun to use MTurk to obtain public opinion on health care-related topics [10].

Our group has worked to understand the relative strengths and weakness of various patient engagement activities for research prioritization in the context of low back pain. Low back pain occurs in 80% of the population at some point in their life [11], accounting for about 8% of all disability from all disease in the United States; 25% of the population reports having had back pain in the past 3 months and 55% report back pain in the past year [12,13]. Despite its prevalence and health burden, there is no clear mechanism for patient engagement in the decision making around back pain research [5]. In a prior study, we compared the research priorities established by patients with back pain who participated in a patient registry with those established by MTurk participants who self-reported having back pain. The 2 groups ranked research topics similarly, despite large differences in age (the MTurk cohort being younger) and in selection into the cohorts: those in the patient registry had a formal diagnosis of back pain, whereas the MTurk group was selected on the basis of their Roland Morris Disability Questionnaire (RMDQ) score. The RMDQ is a validated tool that is used to score back-related disability and was used as a proxy to distinguish those with back pain from those without back pain. The conclusion of the study was that these two methods of identifying patients for engagement—patient registries and crowdsourcing—complement one another [14].

Our prior study exposed difficulties in participant selection from a crowdsourced sample for research prioritization. We had used the RMDQ to find those with back pain but had no understanding of whether this selection process changed the ranking of research topics or improved the information gathered from our cohort. This study, therefore, expands our prior work to a broader population on MTurk, comparing those who screen positive for back pain against those who screen negative for back pain, with categorization based on the RMDQ score. We sought to understand how these 2 groups differed with respect to their research topic rank lists and additional commentary in order to guide the use of MTurk as a platform to support research prioritization for low back pain. We hypothesized that this comparison would also give insight into the use of MTurk for research prioritization, generally.

## Methods

### Overview

**Figure 1:**
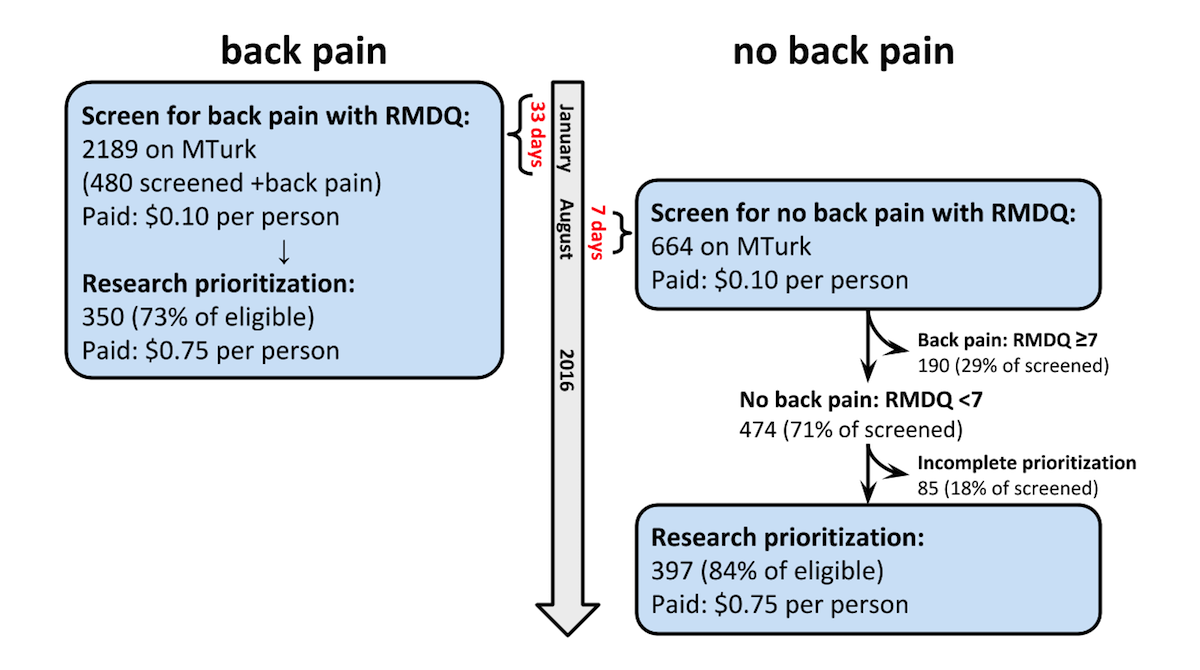
Mechanical Turk (MTurk) enrollment. Schematic flow diagram of enrollment of both cohorts, including screening and response rates. Compensation is in US $. See also Figure 1 in Truitt et al [15]. RMDQ: Roland Morris Disability Questionnaire.

This study is part of a series of investigations to understand methods of patient engagement, and specifically research topic prioritization for back pain [14-16]. We conducted 2 cross-sectional surveys via MTurk: the first in January 2016 targeting those with back pain, and the second in August 2016 targeting those without back pain (Figure 1), limiting the MTurk sample to only those residing in the United States. The University of Washington Human Subjects Division provided ethical approval for this study prior to administration of the surveys.

We used the RMDQ as a screen to categorize individuals as those with back pain (RMDQ ≥ 7) and without back pain (RMDQ < 7) [17]. The RMDQ screens for current back-related disability but does not offer clear insight into a possible history of back pain. Therefore, the group without back pain could have contained individuals with a history of back-related disability that had since improved. We paid MTurk users US $0.10 for completing the RMDQ. We invited a subset of those who took the screening survey to complete a prioritization activity, based on the above categorization. The prioritization survey was extended to those with back pain during the first survey administration and to those without back pain during the second administration. This separate prioritization survey elicited participants’ top 5 of 18 back pain research topics adapted from a list previously generated by primary care providers and researchers (see Multimedia Appendix 1) [18]. We paid MTurk users an additional US $0.75 for completing the prioritization survey [18]. In addition, participants could add up to 5 additional topics in open-ended comment fields beyond the topics in the list provided. Users provided demographic information at the conclusion of the prioritization survey. Both the screening RMDQ and the prioritization surveys were administered using Research Electronic Data Capture (REDCap), a software platform specifically designed for electronic data capture in research studies [19]. Both surveys were developed by our team prior to administration as an open survey on MTurk. We used neither randomization nor adaptive questioning methods. We added an internal validation question to the screen such that, if none of the 24 items on the RMDQ applied, participants were instructed to check a box noting this. Those who did not pass this internal check were removed from analysis. Participants were not able to review their answers prior to submitting, but they were able to change answers as they proceeded through screening and prioritization.

### Demographic and Ranking Analysis

We tabulated age, sex, highest level of education attained, current level of employment, and ethnicity and race, reporting frequencies for categorical variables and means for continuous variables to compare participant demographic characteristics. To understand the geographic distribution of our MTurk sample, we tabulated the US states of residences within each group. We created a ranked list of research topics within each group by determining the frequency that each topic was selected as the top 1-5 priorities and ordered them accordingly. A Spearman rank-order coefficient was used to compare the rank lists of research topics generated by each group. A Spearman coefficient close to 1 would signify a high level of agreement in the order of the ranked research topic lists between groups; a value close to 0 would signify little agreement in the rank lists; a value approaching –1 would signify that the rank lists are opposite one another. We performed a Wilcoxon rank sum test, without continuity correction, to understand whether the distribution of rankings—that is, the relative importance of the top- versus bottom-ranked research topic—was the same or different between groups. A significant result (*P*<.05) would indicate that the distributions of rankings are different.

Administering 2 separate surveys at 2 different time points opened the possibility for MTurk users to repeat the exercise. We selected those individuals who took the RMDQ both in January and in August to compare how their RMDQ score changed over the time period and, for those who were eligible to take the prioritization survey twice, how their research prioritizations changed.

### Content and Quality Analysis

We performed a directed content analysis on the additional comments provided by participants in both groups using an iterative process. After reviewing all comments, we generated a list of codes that reflected the content. Two members of our team, blinded to the work of one another, applied the codes and, where there were disagreements, a third member reconciled the code applied. To assess the quality of the content provided through open-ended comments, we created a coding scheme to indicate how helpful comments were for designing future research topics. Those coded as “no information” were comments that were off-topic from back pain and back pain research (eg, “Thanks”). Those coded as “some information” identified a broad topic area, but neither specified further nor gave insight about the study population (eg, “posture” or “cortisone shots”). Finally, those coded as “rich information” identified a broad research topic area and either included a specific research question within that broad topic or gave insight about the study population, or both (eg, “Can the spread of pain be calculated when the first indicators become evident? My pain has spread from the lower lumbar region into the hips and down the legs over the last 25 years.”). We applied all codes using Dedoose version 7.5.9 (SocioCultural Research Consultants, LLC).

## Results

### Overview

We screened a total of 2812 individuals over 40 days. Of those, 718 (25.53%) were grouped as having back pain (RMDQ score ≥7). The prioritization activity was completed by 350 of those with back pain (72.9% of 480 eligible) during the first administration of the screen and by 397 of those without back pain (84% of eligible) during the second administration of the screen.

### Demographic and Ranking Analysis

Table 1 presents the demographic information for the 2 groups.

The groups were similar with respect to age, ethnicity, and race. The 2 groups differed in the proportion of men versus woman, current employment status, highest level of education completed, and marital status (see Table 1). Compared with the US population as a whole, the study cohort from MTurk was younger (US population: 38 years; MTurk cohort: 33 years), had proportionally more women (US population: 51% female; MTurk cohort: 62% female), was more highly educated (US population: 30%; MTurk cohort: 47%), and was less racially diverse (US population: 77% white; MTurk cohort: 81% white) [20]. The study sample represented 48 states and the District of Columbia, with representation from Wyoming and South Dakota missing in the prioritization results.

The rank lists of research topics for the 2 groups were highly correlated (Spearman correlation coefficient, ρ=.88). The 2 groups agreed on 4 of the top 5 and 9 of the top 10 research topics ranked as most important (see Table 2). Those with back pain ranked “treatment—self-care” as their top research topic, whereas those without back pain ranked “diagnosis—causes of back pain” as their top research topic. Both groups ranked topics related to treatment and diagnosis most highly overall, accounting for all of the top 5 most highly ranked topics in the back pain group, and 4 of the top 5 in the no back pain group. The rank lists differed in how the groups ranked the importance of topics such as prevention, clinical definition, and treatment. The Wilcoxon rank sum test was not statistically significant (*P*=.87), indicating a similar distribution of votes for the research topics.

A total of 41 participants (1.45%) took the RMDQ screen twice. Of those, 2 (5%) were eligible to prioritize twice, 33 (81%) maintained the same back pain classification based on the RMDQ cutoff score of 7 to distinguish back pain from no back pain, and 6 (15%) changed from the no back pain group in the first screen to the back pain group in the second screen and were never eligible to participate in the prioritization activity. The mean change in RMDQ score of those who screened twice was 0.7 points (SD 3.5, range –9 to 9). Of the 2 participants eligible to prioritize twice, 1 completed the prioritization activity twice and ranked the same research topic as their top priority both times.

### Content and Quality Analysis

Additional comments were provided by 53 (15.1%) of the group with back pain (n=350) and 44 (11.3%) of the group without back pain (n=397). The comments from the group with back pain were nearly twice as long as comments from the group without back pain as measured by word and character counts (word count average of 17.3 words vs 8.3 words, respectively; see Table 3). The comments from the group with back pain were marginally more informative toward directing future research based on our application of a quality code: only 5% of the comments from the group with back pain were coded as “no information” compared with 17% of the comments from the group without back pain.

**Table 1:** Demographic data, by back pain group.

Characteristics		Back pain (RMDQ^a^ ≥7) (n=350)	No back pain (RMDQ <7) (n=397)	*P* value^b^
Age (years), mean (SD)		36.6 (11.9)	36.1 (12.3)	.36
Total RMDQ score, median (interquartile range)		10 (8-14)	2 (1-4)	N/A^c^
**Sex, n (%)**				.009
	Male	114 (32.8)	166 (42.1)	
	Female	234 (67.2)	229 (57.8)	
**Highest education level, n (%)**				<.001
	Less than high school	6 (1.7)	2 (0.5)	
	High school diploma or equivalent	42 (12.0)	40 (10.1)	
	Some college, no degree	112 (32.1)	97 (24.5)	
	Associate degree	56 (16.0)	38 (9.6)	
	Bachelor’s degree	104 (29.8)	150 (37.9)	
	Professional or graduate degree	29 (8.3)	69 (17.4)	
**Employment status, n (%)**				<.001
	Employed full-time	153 (43.7)	209 (52.9)	
	Employed part-time	74 (21.1)	72 (18.2)	
	Not employed, looking for work	47 (13.4)	48 (12.2)	
	Not employed, not looking for work	23 (6.6)	42 (10.6)	
	Retired	14 (4.0)	19 (4.8)	
	Unable to work	39 (11.1)	5 (1.3)	
**Marital status, n (%)**				.005
	Married	133 (38.0)	179 (45.1)	
	Widowed	8 (2.3)	4 (1.0)	
	Divorced or separated	43 (12.3)	25 (6.3)	
	Single, never married	129 (36.9)	161 (40.6)	
	Living with a partner	37 (10.6)	28 (7.1)	
**Ethnicity, n (%)**				.63
	Hispanic	26 (7.5)	33 (8.5)	
	Non-Hispanic	324 (92.5)	364 (91.5)	
**Race, n (%)**				.31
	American Indian or Alaska Native	2 (0.6)	2 (0.5)	
	Asian	18 (5.1)	30 (7.6)	
	Native Hawaiian or other Pacific Islander	0 (0.0)	1 (0.3)	
	Black or African American	25 (7.1)	25 (6.3)	
	White	286 (81.7)	309 (77.8)	
	Other	4 (1.1)	10 (2.5)	
	Mixed	15 (4.3)	20 (5.0)	

^a^RMDQ: Roland Morris Disability Questionnaire.

^b^Tests of significance were Wilcoxon rank sum test for nonnormally distributed continuous variables (age), and Pearson chi-square test for categorical variables (education, employment, marital status, ethnicity, and race). Race was recategorized into Asian, black or African American, white, and other to perform the test of significance, but the original categories are displayed here. *P*<.05 was considered significant.

^c^N/A: not applicable (no *P* value is reported for RMDQ score, since this was used to divide the groups).

**Table 2:** Ranked (by number of votes) research priorities^a^.

Research topics	Back pain rank (frequency)	No back pain rank (frequency)
Treatment—self-care	1 (176)	2 (213)
Treatment—cost effective	2 (165)	3 (183)
Diagnosis—causes	3 (149)	1 (219)
Diagnosis—effective tests	4 (145)	5 (143)
Treatment—physical health programs	5 (128)	6 (132)
Prevention—disability reduction	6 (120)	4 (163)
Treatment—patient factors predicting good response	7 (115)	7 (130)
Treatment—primary care services	8 (111)	9 (95)
Outcome measures—treatments	9 (105)	10 (91)
Communication—provider education	10 (80)	12 (78)
Communication—patient education	11 (64)	10 (91)
Work and disability—benefits and compensation	11 (64)	15 (54)
Prevention—reduced disability	11 (64)	8 (101)
Treatment—mental health	14 (61)	17 (52)
Communication—evidence dissemination	15 (53)	15 (54)
Work and disability—return to work	16 (49)	14 (60)
Clinical definition—definition of low back pain	17 (47)	13 (62)
Outcome measures—expectations	18 (46)	17 (52)

^a^Research topics were ranked by frequency of being most important (#1 to #5). Rank lists are divided by group (back pain vs no back pain) and ordered by rank of the back pain group.

**Table 3:** Qualitative and quantitative differences in the additional comments between groups (back pain vs no back pain)^a^.

Comparative factors		Back pain (n=350)	No back pain (n=397)	*P* value
**Individual people who commented, n (%)**		53 (15.1)	44 (11.1)	.10
	Total comments, n	95	95	
Average word count		17.3	8.3	<.001
Average character count		99.8	49.9	<.001
**Quality label**^b^				
	“No information:” off-topic, no reference to back pain, n (%)	5 (5)	16 (17)	.02
	“Some information:” identifies a general topic area, but neither specifies a question within a broad topic nor gives context of their comment, n (%)	48 (51)	36 (38)	
	“Rich information:” identifies a general topic and either specifies an area or question within a broad topic or gives insight about the study population, n (%)	42 (44)	43 (45)	

^a^Tests of significance were Wilcoxon rank sum test (word count, character count), chi-square test.

^b^Percentages, noted in parentheses, were calculated as a proportion of the total comments in each group, back pain and no back pain, both of which had 95 comments.

**Table 4:** Topic areas identified by additional comments, by back pain group, subdivided by quality of the comment.

Topic areas	Back pain (RMDQ^a^ ≥7)		No back pain (RMDQ <7)	
	All (n=95)	Topics labeled “rich information” (n=42)	All (n=95)	Topics labeled “rich information” (n=43)
Treatment, n (%)	44 (46)	21 (50)	58 (61)	29 (67)
Communication, n (%)	13 (14)	2 (5)	6 (6)	3 (7)
Prevention, n (%)	11 (12)	6 (14)	3 (3)	1 (2)
No codes applied, n (%)	10 (11)	3 (7)	15 (16)	4 (9)
Epidemiology, n (%)	9 (9)	6 (14)	10 (11)	6 (14)
Diagnosis, n (%)	8 (8)	4 (10)	1 (1)	0 (0)
Work and disability, n (%)	4 (4)	0 (0)	2 (2)	1 (2)
Outcome measures, n (%)	3 (3)	1 (2)	1 (1)	0 (0)

^a^RMDQ: Roland Morris Disability Questionnaire.

We grouped the topic areas of additional comments into 13 overarching categories, some of which are shown in Table 4. Of note, research topics related to treatment were suggested most commonly by both groups, followed by prevention-related topics in the back pain group and epidemiology-related topics in the group without back pain. Considering only the additional comments that were coded as “rich information” (44% from the group with back pain; 45% from the group with no back pain; Table 3), the distribution of topics was largely unchanged (see Table 4).

## Discussion

### Overview

To our knowledge, our work is novel in its use of the MTurk platform for obtaining input on research prioritization and its application of a patient-reported outcome measurement tool to select a cohort from a crowdsourced sample [14]. In fact, only recently has crowdsourcing been used outside of the realm of behavioral and psychological investigations for patient engagement research, and specifically for research prioritization determination [21-23]. The implications of this work are potentially far-reaching: understanding the strengths and limitations of crowdsourcing techniques is important given both the need to engage the public in research activities and the ease of use of platforms such as MTurk.

Obtaining patient and public input and including a diversity of perspectives has posed and remains a challenge. While crowdsourcing platforms can provide a large and often captive audience, finding the right individuals to engage—whether by using a screening survey or by some other method—adds a layer of difficulty. We therefore sought to understand how those with a condition would rank research topics compared with those without a condition. In the context of low back pain, a prevalent condition, the research topic rank lists of those on MTurk with back pain and those without were very similar, with agreement on 4 of the top 5 and 9 of the top 10 topics. However, we found nuanced differences in the ranked lists of research topics and the additional commentary. The groups differed in that those with back pain ranked topics related to treatment as #1 and #2, whereas those with no back pain ranked topics related to diagnosis as their top priority. While the rate at which participants provided additional commentary was similar between groups (15% in the back pain group and 11% in the no back pain group), the level of detail and length of free-text answers differed: those with back pain who provided comments wrote more than twice as much as those without back pain (see Table 3).

In addition, those with back pain provided comments that were longer and of marginally higher quality than those without back pain, and this difference in quality was statistically significant. As compared with traditional methods, MTurk can be used as one method to prioritize research topics in a short time frame. However, given that those without the experience of back pain provided shorter and less content-rich additional comments, a central challenge of using a crowdsourcing method like MTurk will be adequately selecting those participants whose opinions are most representative of the population in question.

### Comparison With Other Approaches for Obtaining Input on Research Priorities

Patient engagement aims to involve those affected by research findings in the research design and implementation process. This study sought to understand the priorities of a broad population through MTurk. Crowdsourcing as an engagement tool could expand the research community’s ability to obtain input throughout the research process, delivering a broad reach to individuals and timely feedback. This study furthers prior work to determine how crowdsourcing could be used for research prioritization, and specifically whom to study [23].

There are no formal criteria by which to evaluate the various types of patient engagement activities [3,24]. How, then, can a team of researchers determine appropriate patient engagement activities for the purpose of research prioritization among the various options available? Those seeking to engage with and learn the opinions of a targeted patient population must weigh several factors in designing outreach and engagement activities, including the ease of implementation, and time and cost requirements; the ability to obtain a representative sampling of opinions from the target population; and the likelihood of those opinions being informative toward answering the overall question. Traditional methods have included focus groups, one-time questionnaires, Delphi technique, voting, and structured group discussion [3]. These methods may be prohibitive due to resource constraints or potentially being nonrepresentative of a target population [25].

In the context of back pain, a prevalent and easily understood condition, we found the rank list of research priorities among those with back pain to be very similar to the rank list among those without back pain. The wide reach of MTurk coupled with its ease of use adds to its appeal. MTurk provides a platform to connect with a broad audience quickly as compared with other traditional survey- or interview-based methods of engagement. While concern exists that MTurk participants can “game the system,” providing false answers in order to earn more and therefore undermining the validity of the data [4], participants provided thoughtful comments about their experiences in our analysis. It seems, then, that for back pain research, crowdsourcing and MTurk are viable patient engagement activities. Future research is needed to explore the relationships between the prevalence of the condition in question and the degree of correlation in research prioritization among those with the condition versus those without.

It must be noted that a core principle of engagement is relationship building [3,26]. The use of MTurk is limited in that the policies of the platform prohibit follow-up communication. Thus, it is limited to more consultative and cross-sectional approaches for obtaining input on research activities. The importance of this point will depend on the purpose of the engagement activity, although as others have advocated, it may be best to view crowdsourcing as a complement rather than a replacement for interviews, in-person meetings, and other conversational techniques [21].

### Limitations

Our study has several key limitations necessary to contextualize our results and conclusions.

First, dividing our study groups using a threshold cutoff of the RMDQ may have yielded a less-specific determination of back pain versus no back pain, meaning that some with a high RMDQ score, and thus a designation of back pain, may not truly have had a medical diagnosis of low back pain. Moreover, the RMDQ identifies those with current back-related disability and, given the prevalence of the disease itself, many of those categorized as having no back pain may have had it in the past, granting insight into the condition. In addition, we performed 2 separate screening surveys during different times of the year, and this could have biased our groupings.

Second, we did not specifically ask individuals about whether they had sought health care for their back pain. Health care utilization and knowledge about health care seeking may be important for some engagement activities and disease topic areas. For this work on back pain, we decided that the opinions and perspectives of people with back pain—regardless of their health care access or utilization—would give valuable and potentially different insights from a sample derived using noncrowdsourced approaches.

Third, there are limitations to generalizing the results derived from an MTurk sampling. Recruitment with MTurk becomes subject to various selection filters that can introduce bias: the MTurk population can vary by time of day and day of the year [7]. Our MTurk study population was younger, more highly educated, less representative of minority races, and with proportionately more females than the US population as a whole [20,27]. Prior studies on how the MTurk population compares with the general population have noted that MTurk participants are younger and more educated, with an overrepresentation of white and Asian races [7]. This makes sense, given the barriers to entering the MTurk market: access to a computer and reliable Internet connection, having a baseline technological literacy, and establishing an online method to receive payment for tasks.

### Conclusion

This work contributes to an understanding of the strengths and weaknesses of using MTurk in patient engagement activities, and specifically research prioritization. MTurk provides a rapid, easy-to-use, and relatively inexpensive method of obtaining public opinion. We found that, while the groups ranked research topics similarly, there were subtle differences in the content and quality of free-text comments. Given these differences, we suggest that supplemental efforts may be needed to augment the reach of crowdsourcing in obtaining the patient’s voice, especially from specific populations.

## Appendix

Multimedia Appendix 1 Mechanical Turk survey including the Roland Morris Disability Questionnaire and prioritization survey.

## Abbreviations

MTurk: Mechanical Turk
RMDQ: Roland Morris Disability Questionnaire
REDCap: Research Electronic Data Capture
