# Supplementary material for: The Promise and Pitfalls of Using Crowdsourcing in Research Prioritization for Back Pain: Cross-Sectional Surveys
Source: J Med Internet Res. 2017 Oct 6;19(10):e341. doi: 10.2196/jmir.8821 (PMC5650676; doi:10.2196/jmir.8821)
Supplement: Multimedia Appendix 1 [file jmir_v19i10e341_app1.pdf]

Back Pain Prioritization Survey

When your back hurts, you may find it difficult to do some of the things you normally do.This list contains sentences that people have used to describe themselves when they have back pain. When you read them, you may find that some stand out because they describe you today, or you may find that none of these statements apply to you.

As you read the list, think of yourself today. When you read a sentence that describes you today, check the box next to it. If the sentence does not describe you, then leave the space blank and go on to the next one. Remember, only tick the sentence if you are sure it describes you today.

(Check all that apply)

- ☐ I stay at home most of the time because of my back.
- ☐ I change position frequently to try and get my back comfortable.
- ☐ I walk more slowly than usual because of my back.
- ☐ Because of my back, I am not doing any of the jobs that I usually do around the house.
- ☐ Because of my back, I use a handrail to get upstairs.
- ☐ Because of my back problem or leg pain, I lie down to rest more often.
- ☐ Because of my back, I have to hold onto something to get out of an easy chair.
- ☐ Because of my back, I try to get other people to do things for me.
- ☐ I get dressed more slowly than usual because of my back.
- ☐ I only stand for short periods of time because of my back.
- ☐ Because of my back, I try not to bend or kneel down.
- ☐ I find it difficult to get out of a chair because of my back.
- ☐ My back is painful almost all of the time.
- ☐ I find it difficult to turn over in bed because of my back.
- ☐ My appetite is not very good because of my back.
- ☐ I have trouble putting on my socks (or stockings) because of the pain in my back.
- ☐ I only walk short distances because of my back.
- ☐ I sleep less well because of my back.
- ☐ Because of my back, I get dressed with help from someone else.
- ☐ I sit down for most of the day because of my back.
- ☐ I avoid heavy jobs around the house because of my back.
- ☐ Because of my back, I am more irritable and bad tempered with people than usual.
- ☐ Because of my back, I go upstairs more slowly than usual.
- ☐ I stay in bed most of the time because of my back.

☐ Please check this box if none of the above sentence describe you today

Based on your responses, you qualify to complete additional survey questions.These questions will ask you to review and prioritize topics for research in back pain.

You will be bonused $0.75 for completing these questions, if you complete them in full and enter you MTurk Worker ID at the end of the survey and in the HIT on the MTurk platform. We anticipate this survey to take you 5-10 minutes to complete. You may also elect to receive only the first $0.10 payment for completing the HIT by selecting "No" below and completing the survey without extra questions.

Below you will find additional information about this part of our study:

Historically, the patient voice has been missing at the early stages of research design when topics and questions are identified. As a result, what matters most to patients may not be reflected in research studies. We are asking for your help to learn how to better involve patients in our work by participating in our survey that will ask you to rank research topics in low back pain.

Although when you answered the initial question, you did not indicate that you had much, if any back pain, we would like to explore what people without back pain identify as important topics for research in back pain. This will help us understand the importance that experience with a disease or condition plays in identifying and prioritizing research topics.

The survey we are asking you to take will list a range of research topics, and you will be invited to rank topics. We will also ask you to complete a brief survey about yourself, such as information about your back pain (or lack of back pain), and your age, gender, education, etc. We will not be collecting any information that could identify you in any way. We will use the information you provide to us about yourself to generally describe study participants.

The risks of participating in this study include a potential loss of confidentiality, inherent to being on the Amazon MTurk platform. We work to minimize this risk by not collecting any information that could identify you to us. We will be collecting your MTurk Worker ID, but we have no way of connecting that ID to any personally identifying information about you. If any question makes you uncomfortable, you can choose not to answer it. You may also end your participation in the study at any time.

You may not directly benefit from taking part in this research study. We hope the results of this research study will help us know how to best involve patients in the early stages of medical research.

Are you interested in completing additional questions for a bonys payment opportunity?	◯ Yes<br>◯ No

Topics Prioritization Questionnaire

Research TopicsWe invite you to select 5 topics from those listed in the tables below that you think are the most important for back pain researchers to study. Then rank those 5 topics in order of importance. If you feel that there are important research topics that do not appear on the attached list, you may identify up to an additional 5 research needs again in order of priority.

Low Back Pain Research Topics: A list of topics identified by clinicians for further research, with examples of how findings could inform care, is listed as follows. Please review this list and indicate the topics you feel are most important given your experience with back pain.

ID #	Topic Category	Topic	Examples of How Research Findings Could Help Improve Patient Care
1	Clinical Definition	How should low back pain be defined?	Help create a common definition of low back pain for patients, doctors and clinicians.
2	Communication	What are the best ways to provide information to patients with low back pain in primary care?	Help doctors understand how best to provide information to patients about back pain. For example, through brochures, internet resources, or in-person consultation.
3	Communication	What strategies are best for educating doctors to improve their communication & counseling skills regarding low back pain?	Help create training for doctors to improve how they communicate with patients about back pain and treatments.
4	Communication	What are the best strategies for putting research results into clinical practice?	Help doctors know about and access high quality and recent research findings for treating low back pain.
5	Diagnosis	What are effective tests to diagnose low back pain?	Help doctors better understand what tests are helpful diagnosing patients with low back pain.
6	Diagnosis	What are the causes of low back pain?	Help doctors and patients understand all of the possible causes for and continuation of low back pain.
7	Prevention	How can the burden of low back pain disability be reduced?	Help doctors and patients understand what leads to disability caused by low back pain and how to prevent it.
8	Prevention	What are the most effective ways to reduce work disability due to low back pain?	Help inform employers and employees about workplace activities to prevent work disability due to low back pain.
9	Treatment	What patient factors predict a good response to treatment for low back pain?	Help doctors tailor treatments for individual patients. For example, research may find that women may respond to certain types of treatment as compared to men.
10	Treatment	How can we best organize our primary care services to become more efficient in helping patients with low back pain?	Help learn how to organize care so patients receive necessary and timely treatment for low back pain with minimal barriers.
11	Treatment	How can physical health programs be improved to help people experiencing low back pain?	Findings from this research would help doctors understand how to use other aspects of health (such as physical therapy, yoga, exercise) to help relieve symptoms or improve quality of life for people with low back pain.
12	Treatment	How can we improve self-care strategies for low back pain?	Help patients use self-care strategies in addition to their doctor-prescribed treatment plans.
13	Treatment	What are the most cost-effective treatments for low back pain?	Help inform doctors and patients about what treatments would be least expensive while still being effective in terms of reducing low back pain.
14	Treatment	How can mental health programs be improved to help people experiencing low back pain?	Findings from this research would help doctors understand how to use other aspects of health and well-being (such as mental health or cultural programs) to help relieve symptoms or improve quality of life for people with low back pain.
15	Outcome Measures	What are the most important measures of success for low back pain treatments?	Help ensure that the effects of treatments are measured in ways that both doctors and patients think are important.
16	Outcome Measures	How do patient and provider opinions, beliefs, and expectations influence outcomes of care for low back pain?	Help improve results for low back pain treatment by incorporating knowledge about patient beliefs, values, and expectations into treatment plans for low back pain.
17	Work and Disability	What can be done to encourage patients with low back pain to return to work?	Help evaluate workplace initiatives or legislation designed to better support the needs of employees with low back pain.
18	Work and Disability	How do benefit systems (such as compensation or social security disability) influence care received for low back pain?	Help find improvements to benefits systems and insurance design to improve patient care and outcomes in low back pain.

Instructions for ranking:1. Choose 5 topics from the above list of low back pain research topics that you think are most important. 2. Place the ID # for the topic in the column titled "Chosen 5 Topics" in the table below. List the topics in order of importance. Priority 1 indicates the most important topic and Priority 5 indicates the fifth most important topic of your selections. Please do not list a given topic more than once.3. If would like to make comments about your selection please do so in the column titled "Comments".

Priority 1 (most important)

____
(Enter one number, 1 through 18, from the above topics)

Priority 1 comments (optional)

____
(We are interested in the reasons for your choices - please tell us here.)

Priority 2 (second most important)

____
(Enter one number, 1 through 18, from the above topics)

Priority 2 comments (optional)

____
(We are interested in the reasons for your choices - please tell us here.)

Priority 3 (third most important)

____
(Enter one number, 1 through 18, from the above topics)

Priority 3 comments (optional)

____
(We are interested in the reasons for your choices - please tell us here.)

Priority 4 (fourth most important)

____
(Enter one number, 1 through 18, from the above topics)

Priority 4 comments (optional)

____
(We are interested in the reasons for your choices - please tell us here.)

Priority 5 (fifth most important)

____
(Enter one number, 1 through 18, from the above topics)

Priority 5 comments (optional)

____
(We are interested in the reasons for your choices - please tell us here.)

If you feel there are topics that should be considered for low back pain research but are missing from the list provided, please write them in the table below in order of importance to you. You may identify up to 5 topics (1 = most important; 5 = least important)

Additional topic 1:

____
(optional)

Additional topic 2:

____
(optional)

Additional topic 3:

____
(optional)

Additional topic 4:

____
(optional)

Additional topic 5:

____
(optional)

Your Demographic Information

1. Age:

____
(years)

2. Gender:

◯ Male
◯ Female

3. Education level:
(please check your highest level of education)

◯ Less than high school
◯ High school diploma or equivalent
◯ Some college, no degree
◯ Associate's degree
◯ Bachelor's degree
◯ Master's degree
◯ Doctoral or professional degree

4. Employment Status:
(please select one)

◯ Employed full-time
◯ Employed part-time
◯ Not employed, looking for work
◯ Not employed, NOT looking for work
◯ Retired
◯ Unable to work

5. Marital Status?
(please select one)

◯ Married
◯ Widowed
◯ Divorced
◯ Separated
◯ In a domestic partnership or civil union
◯ Single, never married

6. Ethnicity:
(please select one)

◯ Hispanic or Latino
◯ Not Hispanic or Latino

7. Race:
(please select all that apply)

☐ American Indian or Alaska Native
☐ Asian
☐ Native Hawaiian or other Pacific Islander
☐ Black or African American
☐ White
☐ Other

7.1 Race - Other, please explain:

____

8. State of Residence

◯ AL Alabama
◯ AK Alaska
◯ AZ Arizona
◯ AR Arkansas
◯ CA California
◯ CO Colorado
◯ CT Connecticut
◯ DE Delaware
◯ DC District of Columbia
◯ FL Florida
◯ GA Georgia
◯ HI Hawaii
◯ ID Idaho
◯ IL Illinois
◯ IN Indiana
◯ IA Iowa
◯ KS Kansas
◯ KY Kentucky
◯ LA Louisiana
◯ ME Maine
◯ MD Maryland
◯ MA Massachusetts
◯ MI Michigan
◯ MN Minnesota
◯ MS Mississippi
◯ MO Missouri
◯ MT Montana
◯ NE Nebraska
◯ NV Nevada
◯ NH New Hampshire
◯ NJ New Jersey
◯ NM New Mexico
◯ NY New York
◯ NC North Carolina
◯ ND North Dakota
◯ OH Ohio
◯ OK Oklahoma
◯ OR Oregon
◯ PA Pennsylvania
◯ RI Rhode Island
◯ SC South Carolina
◯ SD South Dakota
◯ TN Tennessee
◯ TX Texas
◯ UT Utah
◯ VT Vermont
◯ VA Virginia
◯ WA Washington
◯ WV West Virginia
◯ WI Wisconsin
◯ WY Wyoming

Amazon Mechanical Turk ID

What is your Amazon Mechanical Turk Worker ID?

____

(Please make sure that this number matches the number you enter into the MTurk HIT before submitting. If these numbers do not match, we will reject your assignment, and you will not receive payment.)

Thank you for your participation!
